# Effectiveness of hospital emergency department regionalization and categorization policy on appropriate patient emergency care use: a nationwide observational study in Taiwan

**DOI:** 10.1186/s12913-020-06006-7

**Published:** 2021-01-06

**Authors:** Chih-Yuan Lin, Yue-Chune Lee

**Affiliations:** 1 Department of Neurology, Taipei City Hospital, Taipei, Taiwan; 2 Institute of Health and Welfare Policy, School of Medicine, National Yang-Ming University, Taipei, Taiwan; 3 Master Program in Trans-disciplinary Long-Term Care and Management, National Yang-Ming University, Taipei, Taiwan; 4 Department of Health Care Management, National Taipei University of Nursing and Health, Taipei, Taiwan

**Keywords:** Emergency care, Utilization, Appropriate, Regionalization, Categorization, Policy, NYU-ED algorithm, Observational study

## Abstract

**Background:**

Emergency department (ED) overcrowding is a health services issue worldwide. Modern health policy emphasizes appropriate health services utilization. However, the relationship between accessibility, capability, and appropriateness of ED use is unknown. Thus, this study aimed to examine the effect of hospital ED regionalization policy and categorization of hospital emergency capability policy (categorization policy) on patient-appropriate ED use.

**Methods:**

Taiwan implemented a nationwide three-tiered hospital ED regionalization and categorization of hospital emergency capability policies in 2007 and 2009, respectively. We conducted a retrospective observational study on the effect of emergency care policy intervention on patient visit. Between 2005 and 2011, the Taiwan National Health Insurance Research Database recorded 1,835,860 ED visits from 1 million random samples. ED visits were categorized using the Yang-Ming modified New York University-ED algorithm. A time series analysis was performed to examine the change in appropriate ED use rate after policy implementation.

**Results:**

From 2005 to 2011, total ED visits increased by 10.7%. After policy implementation, the average appropriate ED visit rate was 66.9%. The intervention had no significant effect on the trend of appropriate ED visit rate.

**Conclusions:**

Although regionalization and categorization policies did increase emergency care accessibility, it had no significant effect on patient-appropriate ED use. Further research is required to improve data-driven policymaking.

**Supplementary Information:**

The online version contains supplementary material available at 10.1186/s12913-020-06006-7.

## Background

The American Medical Association and American College of Emergency Physicians published guidelines for the categorization and regionalization of hospital-based emergency department (ED) capability to integrate network emergency care and match critically ill patients with the appropriate healthcare facility [1–3]. Kocher et al. [4] proposed definitions and a conceptual framework for the categorization, designation, and regionalization of emergency care policy evaluation criteria. These theoretical guidelines significantly influence the development of emergency care delivery systems worldwide.

The categorization policy focuses on all emergency care capability and capacity of a hospital, whereas the regionalization policy matches medical resources to patients’ needs to maximize health benefits and outcomes while minimizing the cost and use of resources over a specified geographic area [5]. Empirical data regarding emergency care categorization [5–13] and regionalization [14–21] have demonstrated their effectiveness. However, most studies focus on specific time-sensitive disease entities, age groups, or regional scales. Moreover, data show that categorization, designation, and regionalization policies may improve the transparency of emergency care services by disclosing quality information about the capability levels of prehospital emergency medical services, EDs, and hospital emergency services, but changes in health care-seeking behavior as a result of categorization and regionalization information are unclear [4].

ED overcrowding is a health services research issue [22–26] that may profoundly jeopardize patient safety [27–32]. According to the Asplin ED crowding input-throughput-output conceptual model, patient flow management may drive ED crowding research [33]. This model proposed that ED crowding may be caused by (1) the input dimension represented by inappropriate ED use by patients, and (2) inadequate accessibility and emergency physician capability in emergency care supplied by providers [33].

Aday and Andersen noted that health policy may improve access, thus increasing realized utilization [34]. Contemporary health policy is driven by an emphasis on appropriate health services utilization, avoiding overuse, misuse, or underuse [35]. However, the relationship between accessibility, capability, and appropriateness of ED use remains unknown. Therefore, we hypothesized that regionalization and categorization policies may increase user access, and emergency care quality and capability information disclosure may guide patients when choosing an “appropriate” treatment setting, thereby improving appropriate ED care use rate. Thus, this study aimed to investigate the effect of these policies on patient-appropriate ED use.

## Methods

### Setting

The Taiwan Ministry of Health and Welfare (MoHW) adopted the categorization, designation, and regionalization international guidelines to establish a national emergency care system, including prehospital emergency medical services (EMS), emergency medical responder-responsive hospital designation, regionalization, and categorization of hospital emergency capability. Taiwan’s ED regionalization and categorization policies were implemented in 2007 and 2009, respectively, with the primary goals of establishing infrastructure and decreasing regional disparity.

### Study design and timelines

**Fig. 1:**
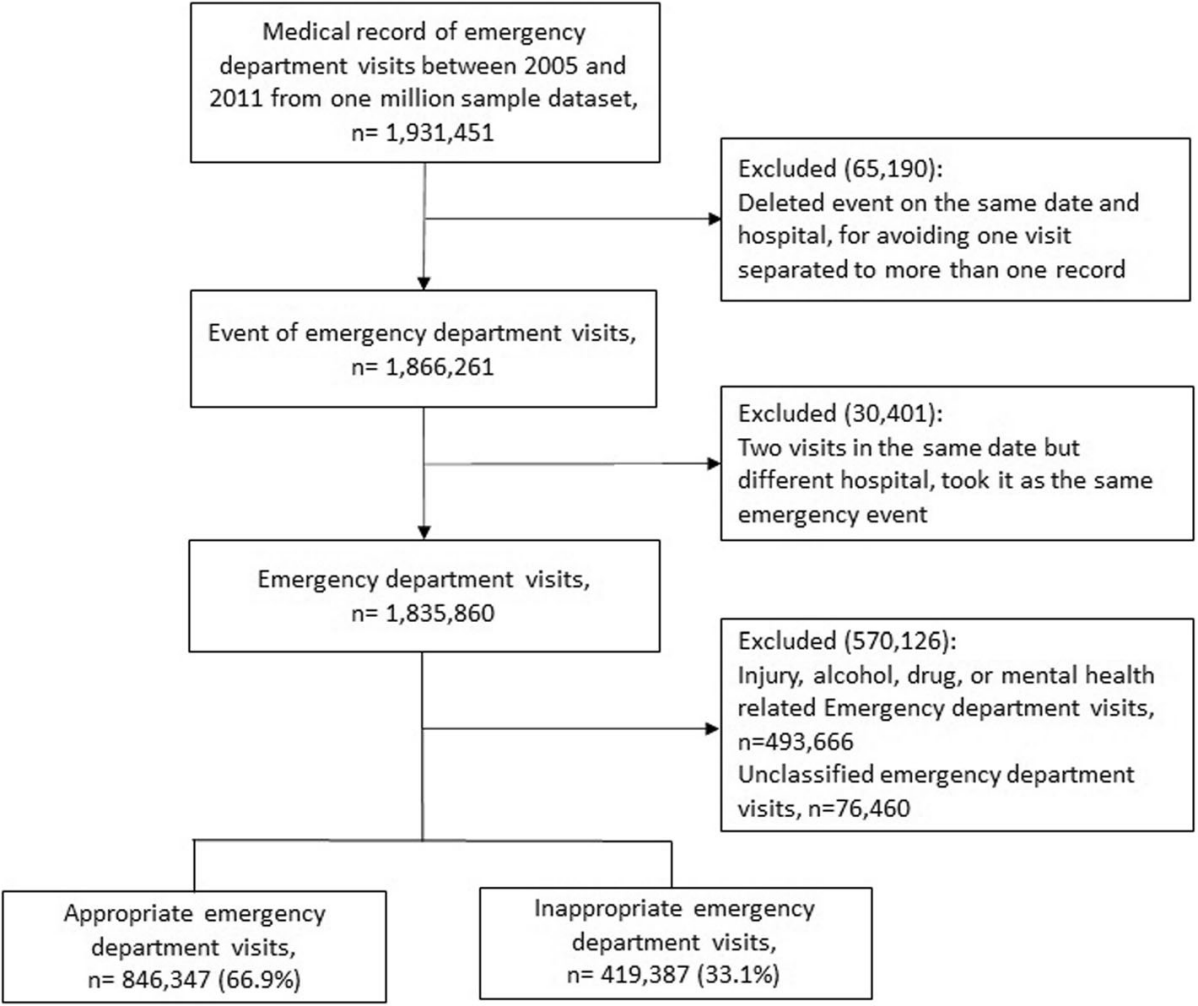
Flow chart of emergency department visits

This seven-year retrospective observational study on the effect of hospital emergency care policies on patient-appropriate ED use included all ED visits between January 1, 2005 and December 31, 2011. Each visit date was associated with one event to avoid replicating events (Fig. 1). We divided the study period in three: the pre-policy period (August 2005–July 2007), regionalization policy intervention period (August 2007–July 2009), and categorization policy intervention period (August 2009–July 2011).

### Regionalization policy

The regionalization policy focuses on prehospital EMS networking and rescuer-responsive hospital ED designations. Its primary objective was to increase the accessibility of emergency care. To achieve this objective, health authorities first identified responsible hospitals in 61 subregions; subsequently, every subregion had at least one hospital that was designated to respond to acute events.

In July 2007, Taiwan launched the Emergency Medical Services Act Amendment. The revised Emergency Medical Services Act represented a landmark for reorganizing the network of emergency care systems in Taiwan; this act implemented three significant changes: (1) introducing hospital-based ED categorization focused on ED medical capability to provide resuscitation, (2) establishing a medical director system to connect hospital ED physicians with prehospital EMS, and (3) forming six regional emergency operations centers (REOCs). Prehospital regionalization efforts included onsite auditing by the MoHW and the categorization of hospital-based ED using a three-tiered approach to emergency care (severe, moderate, and general). Moderate- and severe-tiered EDs undergo onsite audits by the MoHW, while general-tiered hospitals are overseen by the local health authority.

The hierarchical system of emergency rescuer-responsive hospitals and designated hospital ED must provide transport within 30 min for time-sensitive events such as stroke, acute myocardial infarction, major trauma, high-risk pregnancies, and newborns. Six REOCs may dispatch EMS ambulances and rescue personnel from local hospitals to emergency patients and mass casualties. The REOC coordinates with the EMS to provide onsite prehospital triage and care, and dispatches patients to the nearest designated appropriate hospital ED.

### Categorization policy

In 2009, the Taiwan MoHW promulgated the “Standards for the categorization of hospital emergency capability.” According to these standards, the MoHW categorized hospitals into three tiers (severe, moderate, and general) based on their emergency care capability and capacity (including hospital ED, critical time-sensitive-event care teams, and intensive care unit). The primary focus of this policy was the establishment of centers for trauma, cardiac catheterization, stroke, perinatal emergency care conditions, high-risk pregnancies, and pediatric intensive care.

The Joint Commission of Taiwan accredits hospitals every 3 years, and severe-tiered hospitals are considered the last line for hospital referrals. At the end of 2009, 191 rescuer-responsive hospitals were accredited, including 26 severe-, 76 moderate-, and 89 general-tiered hospitals. This policy ensures the use of timely, continuous, effective, and collaborative multidisciplinary methods with two principal objectives: (1) to provide quality hospital emergency care, and (2) to provide a hospital emergency care capability classification with information regarding whether patients chose the appropriate treatment setting (right care in the right place). This information provides patients with appropriate quality information to increase the appropriate ED use. Therefore, the prehospital EMS, hospital ED, hospital emergency care teams, and intensive care unit critical care capability and capacity were well established and categorized in Taiwan [36].

### Estimated appropriate ED use

We used a revised version of the Modified Billings/New York University-ED algorithm (NYU-ED algorithm) for emergency care evaluation according to processes and outcome indicators—referred to as the Yang-Ming modified NYU-ED algorithm—to define the appropriateness of ED use (Additional Fig. 1) [37]. This algorithm classifies ED visits as emergent vs. nonemergent, and optimal care site as ED vs. primary care. It further divides an ED visit into six categories: (1) “nonemergent” (NE), (2) “emergent, primary care treatable” (EPC), (3) “emergent care needed, preventable/avoidable” (EPA), (4) “emergent care needed, not preventable/avoidable” (ENPA), (5) unclassifiable, and (6) “injury and behavioral health-related diagnosis” [38].

The Modified Billings/NYU-ED algorithm classifies ED use as appropriate when EPA and ENPA summative primary diagnosis probabilities are equal to or greater than 0.50, whereas NE and PCT are classified as inappropriate ED use [39–41].

We reclassified inappropriate and unclassifiable ED visits using explicit procedures (if a patient received any procedure that is not often available in primary care settings, such as computed tomography or magnetic resonance imaging scans), and outcome-based criteria (if a patient is transferred, hospitalized, or dies) to reflect the complexity of a patient’s condition and appropriateness of emergency care usage. Injury and behavioral health ICD-9 codes were excluded as the original Billings/NYU-ED algorithm [38].

### Data access and covariates

The analytic data were derived from the Taiwan Longitudinal Health Insurance Database 2005 (LHID2005), which is maintained with routinely collected administrative data from the National Health Insurance [42, 43]. The LHID2005 comprises 1 million randomly sampled participants who were alive in 2005; the database includes all medical records for these individuals from 1995 onward. It also includes hospital and medical professional staff characteristics, subject enrollment, and medical information. This database reflects healthcare facilities’ accessibility and medical care utilization [44, 45]. The collected medical utilization information includes diagnosis procedure, treatment medication, medical cost, date of visit, and deposition [46]. The accuracy of the diagnoses recorded by the LHID2005 for time-sensitive diseases, such as acute ischemic stroke [47, 48], acute myocardial infarction [48], asthma [49] and pneumonia [50], has been validated. Encrypted unique personal identification numbers link all the data, allowing a longitudinal follow-up.

We identified ED visits using the ED visit case type and revenue codes. We used these data to count ED visits. We obtained visit-level data for all ED visits to all hospitals in Taiwan from 2005 to 2011. The covariates included predisposing factors such as sex, age, and occupation; enabling factors such as the beneficiary’s insured salary, urbanization of living area, and emergency care resources classification; and need-driven factors such as comorbidity status. Comorbidity status was defined using the Charlson Comorbidity Index (CCI) score [51].

### Statistical analyses

We compared the two policy periods’ baseline covariates characteristics using χ^2^ tests and t-tests accounting for the LHID2005 administrative data. Because ED use and its covariates are time-dependent and longitudinal, we performed a time series analysis to estimate multilevel changes in appropriate ED use rates after regionalization and categorization policy interventions. Before examining the policy’s effect on appropriate ED visit rates, the Dickey-Fuller test was used to determine the baseline appropriate use rate. The difference in monthly mean probabilities after hospital ED regionalization and categorization was determined using a segmented autoregressive integrated moving average (ARIMA) model with an indicator variable for the regionalization and categorization periods. This model can examine the policy intervention effect while accounting for autocorrelation and time effects. We also evaluated the odds of having an appropriate ED visit during the regionalization and categorization policy stage compared with previous policy stage. All analyses were conducted using SAS 9.4 (SAS Institute, Cary, NC) and Stata MP 14.0 (StataCorp LLC, College Station, TX) software. Statistical significance was determined using a two-tailed significance level of 0.05.

### Sensitivity and Bias analyses

According to a systematic review on ED crowding, the common causes of input factors are nonurgent visits, frequent flyers, and influenza season [52]. During our study period, no specific ED-related policy was promulgated nor was the ED co-payment scheme changed. We evaluated the effects of frequent ED users (≥ 4 visits per year) [53] (Additional Fig. 2) and the 2009 influenza season (Additional Fig. 3) [54]. Excluding the effects related to these factors, the trend in appropriate ED visit classification showed no substantial changes. A sensitivity analysis was conducted increasing the threshold of EPA and ENPA summation probabilities to ≥0.75 (Additional Fig. 4). Similar trends in ED visit classification further demonstrated the lack of significant changes.

## Results

### Emergency Care access

From 2005 to 2011, the number of ED visits, ED expanse, and ED physicians increased by 10.7, 29.4, and 35.1%, respectively. In contrast, the total number of hospitals and EDs decreased by 8.1 and 8.6%, respectively, during the same period (Additional Table 1).

### Participant characteristics

Between 2005 and 2011, 1,931,451 ED visits were identified from 1 million random sample datasets. Thus, the study sample comprised 1,866,261 events (Fig. 1). Of these, 65,190 events on the same date and at the same hospital were excluded to avoid visit replication. When two visits occurred on the same date but at different hospitals (30,401 cases), both visits were considered the same emergency event. A total of 76,460 visits related to injury and behavioral ICD-9 diagnoses were excluded from the modified Billings/NYU-ED algorithm for international comparison. In this study cohort, the crude appropriate ED visit rate was 66.9%. Table 1 presents the numbers and percentages of baseline characteristics of ED visits by policy intervention for observable samples. During the pre-policy period, the mean age of patients was 39.6 ± 26.2 years; after regionalization policy implementation, 44.8 ± 25.3 years; and after categorization policy implementation, 46.2 ± 24.5 years. During the pre-policy period, the mean CCI score of patients was 1.0 ± 1.9; after regionalization policy implementation, 1.2 ± 2.1; and after categorization policy implementation, 1.2 ± 2.1.

**Table 1:** The baseline characteristic among the emergency department visit patients by policy intervention

	Regionalization Policy Intervention			Categorization Policy Intervention		
	Pre-policy period (***n*** = 475,991)	Regionalization period (***n*** = 340,870)		Regionalization period (***n*** = 340,870)	Categorization period (***n*** = 448,873)	
	n (%)	n (%)	***P*** Value	n (%)	n (%)	***P*** Value
**Sex**						
Female	*240,804 (50.6)*	173,685 (51.0)	0.034	173,685 (51.0)	228,441 (50.9)	0.72
Male	235,187 (49.4)	167,185 (49.0)		167,185 (49.0)	220,432 (49.1)	
**Age**						
< 18	116,178 (24.4)	58,052 (17.0)	< 0.001	58,052 (17.0)	65,081 (14.5)	< 0.001
18–64	251,361 (52.8)	189,921 (55.7)		189,921 (55.7)	261,301 (58.2)	
≥ 65	108,452 (22.8)	92,897 (27.3)		92,897 (27.3)	122,491 (27.3)	
**Charlson Comorbidity Index**						
CCI ≤ 1	371,818 (78.1)	251,609 (73.8)	< 0.001	251,609 (73.8)	328,616 (73.2)	0.002
CCI > 1	104,173 (21.9)	89,261 (26.2)		89,261 (26.2)	120,257 (26.8)	
**Place of residence**						
Urban	119,532 (25.1)	86,117 (25.3)	0.11	86,117 (25.3)	114,076 (25.4)	0.58
Suburban	147,321 (31.0)	105,668 (31.0)		105,668 (31.0)	138,697 (30.9)	
Rural	202,969 (42.6)	145,196 (42.6)		145,196 (42.6)	190,850 (42.5)	
Missing	6169 (1.3)	3889 (1.1)		3889 (1.1)	5250 (1.2)	
**Place of ED resources**						
Sufficient area	385,958 (81.1)	277,692 (81.5)	0.005	277,692 (81.5)	364,120 (81.1)	0.012
Insufficient area	90,033 (18.9)	63,178 (18.5)		63,178 (18.5)	84,753 (18.9)	
**Day of visit**						
Weekday	306,106 (64.3)	220,022 (64.5)	0.030	220,022 (64.5)	291,980 (65.0)	< 0.001
Weekend	169,885 (35.7)	120,848 (35.5)		120,848 (35.5)	156,893 (35.0)	
**Income level**						
Quintile 1 (Lowest)	163,027 (34.3)	34,632 (10.2)	< 0.001	34,632 (10.2)	45,746 (10.2)	< 0.001
Quintile 2	43,972 (9.2)	105,197 (30.9)		105,197 (30.9)	134,040 (29.9)	
Quintile 3	124,805 (26.2)	85,505 (25.1)		85,505 (25.1)	37,760 (8.4)	
Quintile 4	46,384 (9.7)	40,680 (11.9)		40,680 (11.9)	126,315 (28.1)	
Quintile 5 (Highest)	95,135 (20.0)	70,695 (20.7)		70,695 (20.7)	98,837 (22.0)	
Missing	2668 (0.6)	4161 (1.2)		4161 (1.2)	6175 (1.4)	
**Occupation**						
Dependents of the insured individuals	205,782 (43.2)	128,140 (37.6)	< 0.001	128,140 (37.6)	156,843 (34.9)	< 0.001
Civil servants, teachers, military personnel, and veterans	22,276 (4.7)	24,607 (7.2)		24,607 (7.2)	31,548 (7.0)	
Nonmanual workers and professionals	83,206 (17.5)	60,068 (17.6)		60,068 (17.6)	87,623 (19.5)	
Manual workers	114,506 (24.1)	92,937 (27.3)		92,937 (27.3)	126,101 (28.1)	
Other	47,737 (10.0)	31,090 (9.1)		31,090 (9.1)	40,696 (9.1)	
Missing	2484 (0.5)	4028 (1.2)		4028 (1.2)	6062 (1.4)	

Note: The policy of regionalization started on 13 July 2007. Moreover, the policy of categorization began on 13 July 2009. However, using the 31 July 2007 and 31 July 2009 as the cut of point

### Appropriate ED visit rate by baseline characteristics for different policy interventions

Table 2 presents the overall rate of appropriate ED visits after regionalization policy implementation (64.1% pre-policy period vs. 68.1% regionalization policy stage); no significant changes occurred after the categorization policy was implemented (68.1% vs. 68.5%). Patients with a higher rate of appropriate ED use had the following characteristics: male, aged ≥65 years, CCI score > 1, rural region residents, residents of an area with inadequate emergent care resources, weekday ED visit, income level in the third quintile, and occupations such as civil servants, teachers, military personnel, and veterans.

**Table 2:** Appropriate rate by baseline characteristic on different policy intervention

	Regionalization Policy Intervention, ***n*** = 816,861			Categorization Policy Intervention, ***n*** = 789,743		
	Pre-policy period (***n*** = 475,991)	Regionalization period (***n*** = 340,870)		Regionalization period (***n*** = 340,870)	Categorization period (***n*** = 448,873)	
	No of Appropriate ED Visit (%)	No of Appropriate ED Visit (%)	***P Value***	No of Appropriate ED Visit (%)	No of Appropriate ED Visit (%)	***P Value***
**Total**	305,116 (64.1)	233,970 (68.6)	< 0.001	233,970 (68.6)	307,261 (68.5)	0.17
**Sex**						
Female	153,088 (63.6)	117,849 (67.9)	< 0.001	117,849 (67.9)	154,642 (67.7)	0.38
Male	152,028 (64.6)	116,121 (69.5)	< 0.001	116,121 (69.5)	152,619 (69.2)	0.28
**Age**						
< 18	43,598 (37.5)	21,956 (37.8)	0.29	21,956 (37.8)	23,658 (36.4)	< 0.001
18–64	165,836 (66.0)	129,437 (68.2)	< 0.001	129,437 (68.2)	174,718 (66.9)	< 0.001
≥ 65	95,682 (88.2)	82,577 (88.9)	0.006	82,577 (88.9)	108,885 (88.9)	1.00
**Charlson Comorbidity Index**						
CCI ≤ 1	211,153 (56.8)	153,181 (60.9)	< 0.001	153,181 (60.9)	198,990 (60.6)	0.027
CCI > 1	93,963 (90.2)	80,789 (90.5)	0.21	80,789 (90.5)	108,271 (90.0)	0.078
**Place of residence**						
Urban	72,188 (60.4)	55,220 (64.1)	< 0.001	55,220 (64.1)	72,620 (63.7)	0.13
Suburban	92,955 (63.1)	71,748 (67.9)	< 0.001	71,748 (67.9)	93,557 (67.5)	0.058
Rural	136,783 (67.4)	104,686 (72.1)	< 0.001	104,686 (72.1)	138,021 (72.3)	0.25
Missing	3190 (51.7)	2316 (59.6)	< 0.001	2316 (59.6)	3063 (58.3)	0.32
**Place of ED resources**						
Sufficient area	245,981 (63.7)	189,216 (68.1)	< 0.001	189,216 (68.1)	246,829 (67.8)	0.021
Insufficient area	59,135 (65.7)	44,754 (70.8)	< 0.001	44,754 (70.8)	60,432 (71.3)	0.12
**Day of visit**						
Weekday	207,817 (67.9)	159,223 (72.4)	< 0.001	159,223 (72.4)	210,381 (72.1)	0.042
Weekend	97,299 (57.3)	74,747 (61.9)	< 0.001	74,747 (61.9)	96,880 (61.7)	0.61
**Income level**						
Quintile 1 (Lowest)	105,959 (65.0)	20,879 (60.3)	< 0.001	20,879 (60.3)	27,629 (60.4)	0.79
Quintile 2	29,545 (67.2)	77,534 (73.7)	< 0.001	77,534 (73.7)	98,340 (73.4)	0.13
Quintile 3	85,307 (68.4)	62,461 (73.0)	< 0.001	62,461 (73.0)	25,495 (67.5)	< 0.001
Quintile 4	27,359 (59.0)	26,183 (64.4)	< 0.001	26,183 (64.4)	89,831 (71.1)	< 0.001
Quintile 5 (Highest)	55,478 (58.3)	44,571 (63.0)	< 0.001	44,571 (63.0)	62,849 (63.6)	0.060
Missing	1468 (55.0)	2342 (56.3)	0.36	2342 (56.3)	3117 (50.5)	< 0.001
**Occupation**						
Dependents of the insured individuals	113,133 (55.0)	78,377 (61.2)	< 0.001	78,377 (61.2)	97,312 (62.0)	0.001
Civil servants, teachers, military personnel, and veterans	17,384 (78.0)	19,879 (80.8)	< 0.001	19,879 (80.8)	25,137 (79.7)	0.012
Nonmanual workers and professionals	49,789 (59.8)	37,726 (62.8)	< 0.001	37,726 (62.8)	53,941 (61.6)	< 0.001
Manual workers	88,197 (77.0)	72,606 (78.1)	< 0.001	72,606 (78.1)	97,322 (77.2)	< 0.001
Other	35,280 (73.9)	23,154 (74.5)	0.22	23,154 (74.5)	30,531 (75.0)	0.20
Missing	1333 (53.7)	2228 (55.3)	0.24	2228 (55.3)	3018 (49.8)	< 0.001

Using the chi-squared test to check the percentage of an emergent visit from all emergency department visit by subgroup; *ED* emergency department

### Time series analysis of the policy intervention effect

**Fig. 2:**
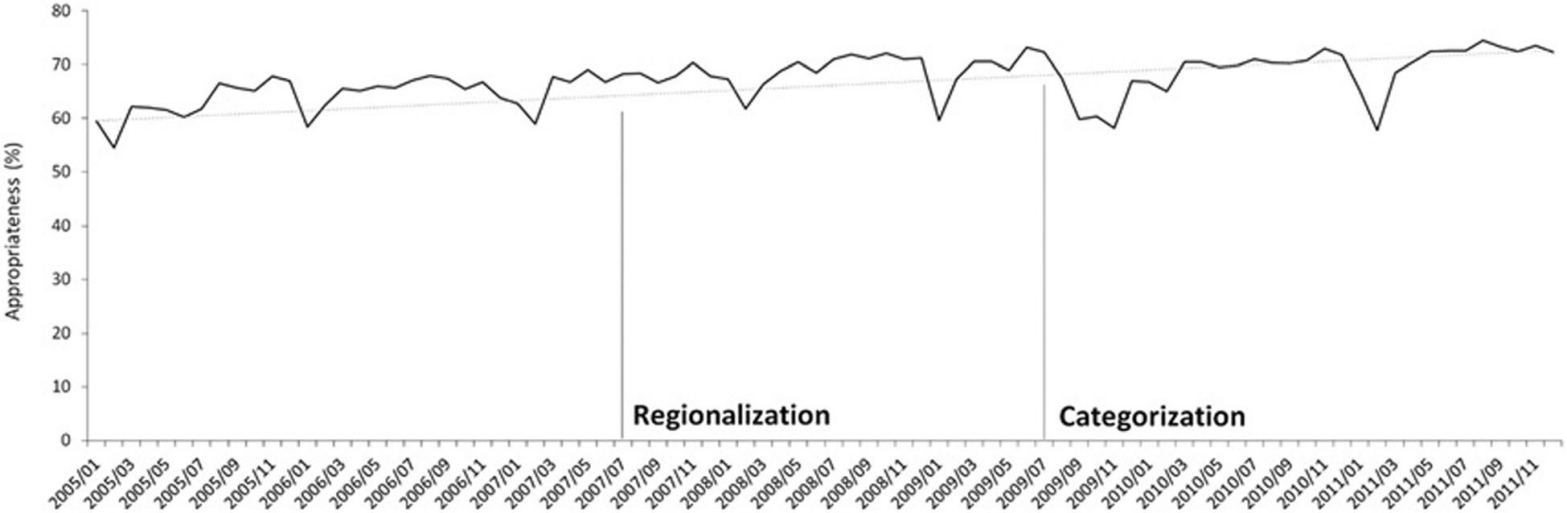
The appropriate rate of emergency department visits during the period from 2005 to 2011 by policies intervention

Figure 2 presents the results of the segmented ARIMA analyses examining the relationship between regionalization and categorization policies and appropriate ED use rate. For the rate of appropriate ED visits during the study period, there was no significant change in intercept points (level parameters). Table 2 presents results showing a statistically significant change in the average appropriate ED visit rate as a result of the regionalization policy. The Dickey-Fuller test showed that the data remained stable before and after the implementation of the regionalization (*P* = 0.005) and categorization (*P* = 0.0037) policies. To fit the ARIMA model, we used the autocorrelation function (ACF) and partial autocorrelation function (PACF) to determine whether autoregressive (AR) or moving average processes terms were needed to address any autocorrelation. The plot of the appropriate ED rate by month showed an exponentially declining ACF and spikes in the first and eleventh lags of the PACF. Therefore, we used the AR term in this study.

We found that comorbidity mediated the association between appropriate ED visit rate and regionalization or categorization policy intervention (Table 3). In contrast, income had an inverse association with appropriate ED use rate. We performed a stratification analysis using the CCI. The population was classified into two groups: one with a CCI score equal to or less than 1 and the other with a CCI score greater than 1 (Additional Table 2). The CCI score subgroup analysis showed that, in both groups, the regionalization policy had a significant negative effect on appropriate ED visit rate (CCI score ≤ 1: trend change − 0.27% per month, *P* = 0.024; CCI score > 1: trend change − 0.16% per month, *P* = 0.015).

**Table 3:** The segmented autoregressive integrated moving average model analysis of the percentage of ED visit

	Regionalization Policy Intervention			Categorization Policy Intervention		
	β	SE	***P*** Value	β	SE	***P*** Value
Baseline trend	**0.06**	**0.05**	**0.25**	**−0.08**	**0.06**	**0.16**
Level change after policy intervention	**−0.79**	**0.74**	**0.28**	**−0.08**	**1.09**	**0.95**
Trend change after policy intervention	**−0.07**	**0.06**	**0.23**	**0.01**	**0.06**	**0.92**
Percentage of female	**0.66**	**0.33**	**0.05**	**0.27**	**0.30**	**0.37**
Mean Charlson score	**30.83**	**2.42**	**< 0.001**	**33.88**	**3.93**	**< 0.001**
Percentage of residence in urban	**0.01**	**0.41**	**0.98**	**0.17**	**0.42**	**0.68**
Percentage of insufficient emergency medical resources area	**0.26**	**0.39**	**0.51**	**0.18**	**0.52**	**0.73**
Percentage of weekend	**0.04**	**0.05**	**0.36**	**0.06**	**0.07**	**0.40**
Percentage of income level at quintile 1 (Lowest)	**0.08**	**0.04**	**0.04**	**−0.19**	**0.95**	**0.84**
Percentage of the dependents of the insured individuals	**−0.13**	**0.14**	**0.35**	**−0.29**	**0.12**	**0.02**
AR1	**0.05**	**0.17**	**0.77**	**−0.04**	**0.19**	**0.82**

*ED* emergency department

## Discussion

In this seven-year policy intervention observational study, ED utilization and expanse increased by 10.7 and 29.4%, respectively. Provider-related ED policies had no significant medium-term effects (2 years) on patients’ patterns of appropriate ED use and improved the realized accessibility to EDs.

Previous studies concluded that appropriate ED utilization varies according to insurance status, socioeconomic status, race, and other sociodemographic factors [7]. Nonetheless, need-driven factors were the most important predictors of appropriate ED use [55]. Conversely, common factors for inappropriate ED use include greater trust in the hospital than in the primary care setting [56], greater convenience [57], time saving [58, 59], or lack of primary care access [60]. One Taiwanese nationwide validation study reported that an increase in the availability of ambulatory care physicians or facilities did not decrease non-emergency ED use [61].

From the user’s perspective, non-business hours, including evening and weekend, accounted for 76.2% of whole service period. Moreover, patients tend to seek immediate or ED services for time-sensitive events [62, 63]. Our study determined that provider-side ED policy implementation and quality information disclosure did not increase patients’ appropriate ED use. The lack of effect may be explained as follows: (1) prudent laypersons may have difficulty judging whether their condition is urgent or nonurgent, and where they should go for treatment [63]; and (2) the categorization of hospital emergency capability ensures the comprehensive availability of laboratory services, image studies, and treatment 24 h a day/365 days a year.

The unintended consequence of hospital emergency care quality disclosure is that the hospital ED becomes the first choice for people seeking treatment for time-sensitive emergency conditions or convenience. The Taiwan MoHW proposed a co-payment of $12 for an ED visit to possibly reduce primary care-treatable ED visits. However, the National Health Insurance medical service payment standards dictate that the co-payments for a medical center ED, regional hospital ED, and local hospital ED visit are $15, $10, and $5, respectively. In comparison, the co-payment for an outpatient clinic visit at a medical center is $12; at a regional hospital, $8; at a local hospital, $2.7; and at a general practitioner office, $1.7. Other factors that must be considered are traffic and waiting time costs in the primary care setting, as well as a minimum wage of $4.50 per hour [64]. Meanwhile, a nationwide emergency policy requires the hospital emergency care system to improve its abilities to meet patients’ needs by improving accessibility through increased convenience (e.g., providing immediate access to an ED anytime) and availability (e.g., providing consultations with an available specialist within 30 min). Therefore, a hospital ED offers more significant time savings and cost-effectiveness for patients.

A systematic review reported that financial constraints and case management are two effective approaches to increase ED use appropriateness [65]. Raven et al. [65] suggested that financial measures may decrease ED visits without increasing appropriateness; conversely, case management may decrease ED visits while increasing appropriateness. We examined the effects of provider-related policy and disclosure of related information on improving patients’ appropriate ED use and found that this policy goal was unmet. We agree with Smulowitz’s suggestion [66] to reshape emergency care and extend medical emergency services to meet patients’ needs, such as offering real-time “face-to-face” telehealth to provide medical recommendations to support patient decision-making, and relieve patient anxiety, implementing an access policy that combines primary and ED care data without time or location limitations [67, 68].

### Strengths and limitations

This study has several strengths: (1) we provided real-world empirical data to explain the relationship between ED policy intervention and patients’ health-seeking behaviors; (2) we analyzed other possible causes of input factor increase (2009 influenza pandemic and frequent ED user effect) to ensure that the effects of policy implementation on appropriate ED use were not caused by other confounders; and (3) to the best of our knowledge, this study is the first nationwide single insurance system example that supports the American Medical Association, American College of Emergency Physicians, and Kocher et al. theoretical models regarding the categorization, designation, and regionalization of emergency care. Thus, our study provides unique information for academic research in emergency care.

Conversely, this study has the following limitations that may impact its generalizability: (1) this retrospective study used a dataset collected for administrative claims purposes according to the conceptualization of appropriate ED visits, which may be defined at the patient, disease, hospital, and social context levels [69–75]. However, we did not have enough information to address these holistic concerns; (2) the LHID2005 administrative dataset is collected for reimbursement purposes, and there is natural attrition due to aging, migration, and death; (3) categorization and designation are essential components in the regionalization of emergency care networks [76]. However, Taiwan emergency care policy and services underwent an established regionalization and categorization sequence, and these paradigm differences deserve further investigation; and (4) in the market-maximized approach, financial and managed care strategies are chosen to drive improvement in appropriate ED use [77]. Contrastingly, Taiwan chose a market-minimized policy to guide appropriate ED use, which may limit the external validity of our study.

## Conclusion

Among policies on international emergency care delivery systems, Taiwan’s promotion of regionalization and categorization of emergency care policies has unique characteristics and requires evaluation. The provider-side changes implemented by these policies did improve patient’s accessibility to emergency care. In contrast, emergency care quality disclosure may not increase patients appropriate ED use. Strategies for balancing patients’ needs and appropriate ED use require further investigation.

## Supplementary information

**Additional file 1: Figure 1.** Yang-Ming modified New York University-Emergency Department Algorithm.

**Additional file 2: Figure 2.** Rate of appropriate emergency department visits excluding frequent users.

**Additional file 3: Figure 3.** Rate of appropriate emergency department visits excluding influenza patients.

**Additional file 4: Figure 4.** Rate of appropriate emergency department visits using a ≥ 0.75 threshold.

**Additional file 5: Table 1.** Regionalization and categorization policy-related statistics in Taiwan.

**Additional file 6: Table 2.** Segmented autoregressive integrated moving average model analysis of the percentage of emergency department visits according to Charlson comorbidity index groups.

**Additional file 7: Table 3**. Segmented autoregressive integrated moving average model analysis of the appropriate ED visit rate by the Charlson Comorbidity Index group.

## Data Availability

The data that support the findings of this study are available from the Taiwan National Health Insurance Research Database, but restrictions apply to the availability of these data, which were used under license for the current study and so are not publicly available. Data are however available from the authors upon academic request and with permission of the Taiwan National Health Insurance Administration.

## Abbreviations

ACF: Autocorrelation function
AR: Autoregressive
ARIMA: Autoregressive integrated moving average
CCI: Charlson Comorbidity Index
ED: Emergency department
EMS: Emergency medical services
ENPA: Emergent, not preventable/avoidable
EPA: Emergent, preventable/avoidable
EPC: Emergent, primary care
MoHW: Ministry of Health and Welfare
NE: Nonemergent
PACF: Partial autocorrelation function
REOC: Regional emergency operations center
