# Supplementary figures and images for: Effectiveness of hospital emergency department regionalization and categorization policy on appropriate patient emergency care use: a nationwide observational study in Taiwan
Source: BMC Health Serv Res. 2021 Jan 6;21:21. doi: 10.1186/s12913-020-06006-7 (PMC7787133; doi:10.1186/s12913-020-06006-7)

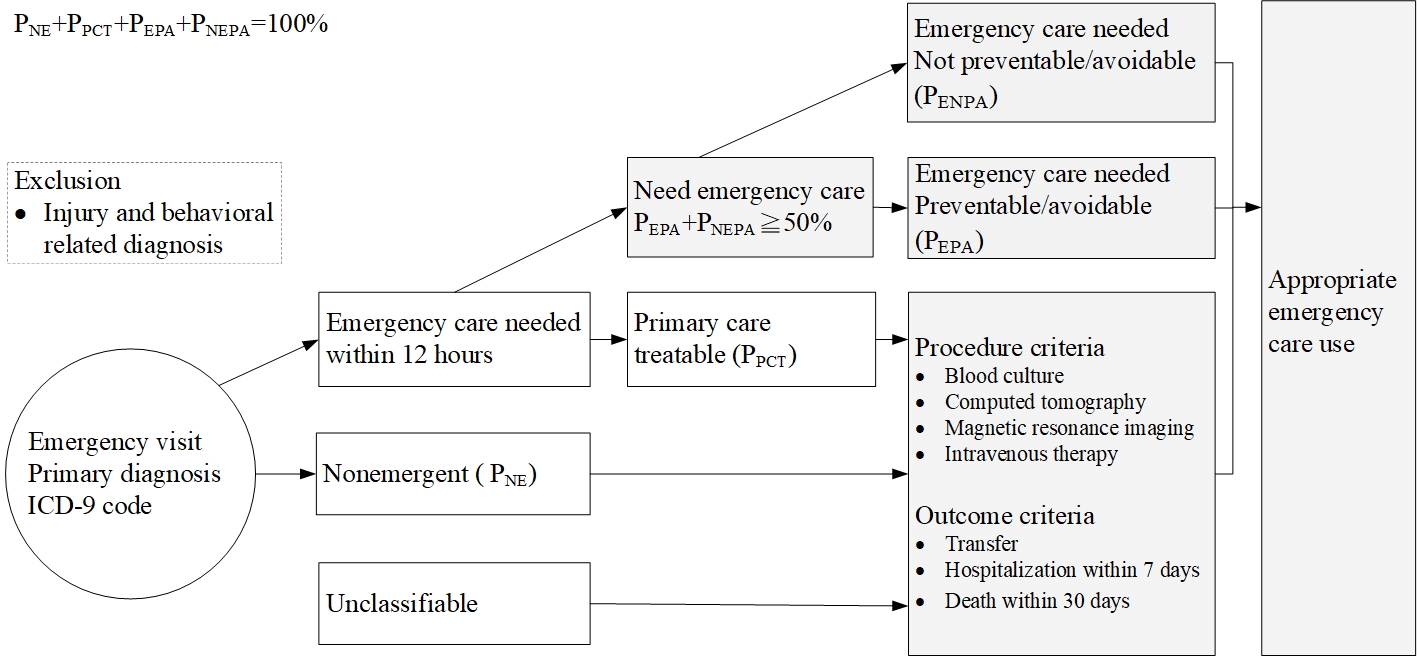

Supplement: Supplementary file 1 — Additional file 1: Figure 1. Yang-Ming modified New York University-Emergency Department Algorithm. [file 12913_2020_6006_MOESM1_ESM.jpg]

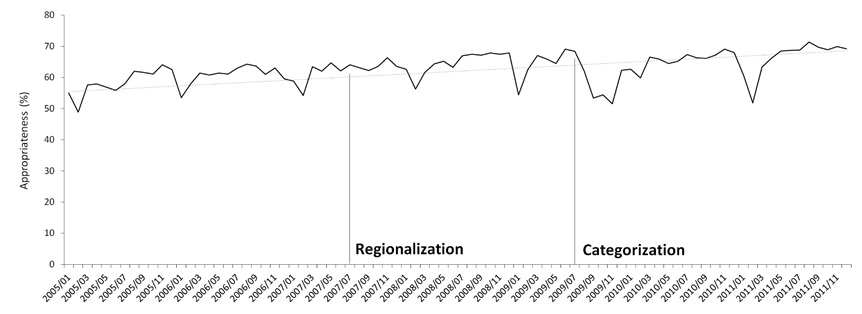

Supplement: Supplementary file 2 — Additional file 2: Figure 2. Rate of appropriate emergency department visits excluding frequent users. [file 12913_2020_6006_MOESM2_ESM.jpg]

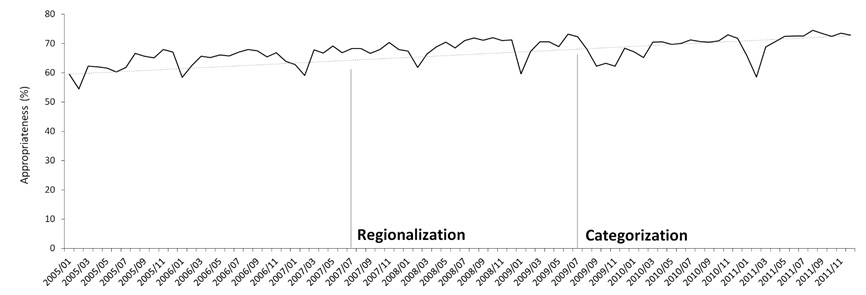

Supplement: Supplementary file 3 — Additional file 3: Figure 3. Rate of appropriate emergency department visits excluding influenza patients. [file 12913_2020_6006_MOESM3_ESM.jpg]

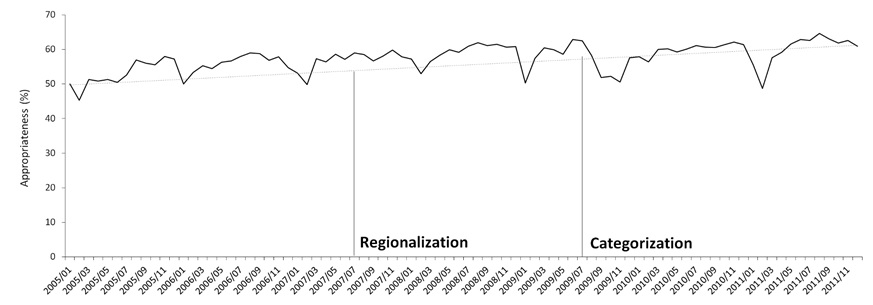

Supplement: Supplementary file 4 — Additional file 4: Figure 4. Rate of appropriate emergency department visits using a ≥ 0.75 threshold. [file 12913_2020_6006_MOESM4_ESM.jpg]
