# Supplementary material for: Effectiveness of hospital emergency department regionalization and categorization policy on appropriate patient emergency care use: a nationwide observational study in Taiwan
Source: BMC Health Serv Res. 2021 Jan 6;21:21. doi: 10.1186/s12913-020-06006-7 (PMC7787133; doi:10.1186/s12913-020-06006-7)
Supplement: Supplementary file 5 — Additional file 5: Table 1. Regionalization and categorization policy-related statistics in Taiwan. [file 12913_2020_6006_MOESM5_ESM.docx]

**Additional Table 1** Regionalization and categorization policy-related statistics in Taiwan unit: thousands person, thousands of visits, million points

| Year | Policy | Level and number of  responsive hospitals | | Population | Hospital | Acute  Bed | Acute bed  occupancy | Physician | ED  Physician | OPD  visits | OPD  expanse | ED  visits | ED  expanse |
| --- | --- | --- | --- | --- | --- | --- | --- | --- | --- | --- | --- | --- | --- |
| 2005 |  |  |  | 22,315 | 531 | 129,548 | 66.3 | 15,398 | 698 | 273,029 | 219,576 | 5,955 | 12,512 |
| 2006 |  |  |  | 22,484 | 523 | 131,152 | 64.6 | 16,152 | 782 | 258,644 | 222,944 | 5,684 | 12,704 |
| 2007 | **Regionalization** | Severe  Moderate General | 13  95  92 | 22,803 | 507 | 131,776 | 65.3 | 16,778 | 900 | 263,166 | 231,535 | 5,798 | 13,630 |
| 2008 |  |  |  | 22,918 | 493 | 133,020 | 66.0 | 17,519 | 947 | 264,503 | 243,689 | 5,700 | 13,966 |
| 2009 | **Categorization** | Severe  Moderate  General | 24  57  109 | 23,026 | 496 | 134,716 | 65.2 | 18,028 | 1,015 | 276,860 | 256,633 | 6,354 | 15,410 |
| 2010 |  |  | | 23,074 | 492 | 135,401 | 66.2 | 18,532 | 1,040 | 282,997 | 265,359 | 6,478 | 16,908 |
| 2011 |  |  | | 23,199 | 491 | 135,431 | 67.8 | 19,061 | 1,076 | 279,852 | 281,298 | 6,671 | 17,731 |
| 2011/  2005 change |  |  | | **3.8%** | **-8.1%** | **4.3%** | **2.1%** | **19.2%** | **35.1%** | **2.4%** | **21.9%** | **10.7%** | **29.4%** |

Source: Ministry of Health and Welfare. The Statistics and Trends in Health and Welfare 2014.

ED: emergency department; OPD: out-patient department.
