# Supplementary material for: Effectiveness of hospital emergency department regionalization and categorization policy on appropriate patient emergency care use: a nationwide observational study in Taiwan
Source: BMC Health Serv Res. 2021 Jan 6;21:21. doi: 10.1186/s12913-020-06006-7 (PMC7787133; doi:10.1186/s12913-020-06006-7)
Supplement: Supplementary file 7 — Additional file 7: Table 3. Segmented autoregressive integrated moving average model analysis of the appropriate ED visit rate by the Charlson Comorbidity Index group. [file 12913_2020_6006_MOESM7_ESM.docx]

**Additional Table 3** The segmented autoregressive integrated moving average model analysis of the appropriate ED visit rate by the Charlson Comorbidity Index group

|  | **Regionalization Policy Intervention** | | |  | **Categorization Policy Intervention** | | |
| --- | --- | --- | --- | --- | --- | --- | --- |
|  | **β** | **SE** | ***P* Value** |  | **β** | **SE** | ***P* Value** |
| **Charlson Comorbidity Index Score ≤ 1** | | | | | | | |
| Baseline trend | 0.26 | 0.10 | 0.008 |  | -0.14 | 0.19 | 0.45 |
| Level change after policy intervention | 0.71 | 1.48 | 0.63 |  | -1.55 | 2.55 | 0.54 |
| Trend change after policy intervention | -0.27 | 0.12 | 0.024 |  | 0.14 | 0.25 | 0.58 |
| Percentage of female | 0.16 | 0.56 | 0.77 |  | -0.09 | 0.82 | 0.92 |
| Percentage of residence in urban | -0.58 | 0.77 | 0.45 |  | -0.16 | 0.89 | 0.86 |
| Percentage of deprivation area | 1.16 | 0.73 | 0.11 |  | 1.29 | 1.50 | 0.39 |
| Percentage of weekend | 0.12 | 0.16 | 0.46 |  | 0.00 | 0.20 | 1.00 |
| Percentage of income level at quintile 1 (Lowest) | 0.20 | 0.08 | 0.015 |  | -2.10 | 1.80 | 0.24 |
| Percentage of the dependents of the insured individuals | -0.71 | 0.24 | 0.003 |  | -0.76 | 0.28 | 0.008 |
| AR1 | -0.01 | 0.19 | 0.96 |  | 0.17 | 0.23 | 0.47 |
| **Charlson Comorbidity Index Score > 1** | | | | | | | |
| Baseline trend | 0.13 | 0.05 | 0.01 |  | -0.01 | 0.04 | 0.75 |
| Level change after policy intervention | 0.29 | 0.96 | 0.76 |  | -1.03 | 0.75 | 0.17 |
| Trend change after policy intervention | -0.16 | 0.06 | 0.015 |  | 0.06 | 0.05 | 0.24 |
| Percentage of female | 0.29 | 0.20 | 0.15 |  | 0.33 | 0.17 | 0.049 |
| Percentage of residence in urban | -0.22 | 0.29 | 0.45 |  | -0.31 | 0.28 | 0.27 |
| Percentage of deprivation area | -0.24 | 0.32 | 0.46 |  | -0.35 | 0.26 | 0.18 |
| Percentage of weekend | -0.07 | 0.08 | 0.38 |  | -0.01 | 0.08 | 0.92 |
| Percentage of income level at quintile 1 (Lowest) | 0.05 | 0.04 | 0.19 |  | 0.52 | 0.68 | 0.45 |
| Percentage of the dependents of the insured individuals | -0.03 | 0.21 | 0.88 |  | 0.24 | 0.23 | 0.30 |
| AR1 | -0.15 | 0.20 | 0.44 |  | 0.03 | 0.20 | 0.89 |

**ED: emergency department.**
